# Recognizing the same face in different contexts: Testing within-person face recognition in typical development and in autism

**DOI:** 10.1016/j.jecp.2015.09.029

**Published:** 2016-03

**Authors:** Louise Neil, Giulia Cappagli, Themelis Karaminis, Rob Jenkins, Elizabeth Pellicano

**Affiliations:** a Centre for Research in Autism and Education (CRAE), UCL Institute of Education, University College London, London WC1H 0NU, UK; b Istituto Italiano di Tecnologia, 16163 Genova, Italy; c Department of Psychology, University of York, York YO10 5DD, UK; d School of Psychology, University of Western Australia, Crawley, Perth, Western Australia 6009, Australia

**Keywords:** Face perception, Face recognition, Autism, Identity, Children, Development

## Abstract

Unfamiliar face recognition follows a particularly protracted developmental trajectory and is more likely to be atypical in children with autism than those without autism. There is a paucity of research, however, examining the ability to recognize the same face across multiple naturally varying images. Here, we investigated within-person face recognition in children with and without autism. In Experiment 1, typically developing 6- and 7-year-olds, 8- and 9-year-olds, 10- and 11-year-olds, 12- to 14-year-olds, and adults were given 40 grayscale photographs of two distinct male identities (20 of each face taken at different ages, from different angles, and in different lighting conditions) and were asked to sort them by identity. Children mistook images of the same person as images of different people, subdividing each individual into many perceived identities. Younger children divided images into more perceived identities than adults and also made more misidentification errors (placing two different identities together in the same group) than older children and adults. In Experiment 2, we used the same procedure with 32 cognitively able children with autism. Autistic children reported a similar number of identities and made similar numbers of misidentification errors to a group of typical children of similar age and ability. Fine-grained analysis using matrices revealed marginal group differences in overall performance. We suggest that the immature performance in typical and autistic children could arise from problems extracting the perceptual commonalities from different images of the same person and building stable representations of facial identity.

## Highlights

• We examined the ability to recognise the same face across varying images.
• Participants were given 40 photographs of two distinct identities and asked to sort them by identity.
• Children divided images into more perceived identities than adults.
• Children with autism performed similarly to typically developing children.
• Within-person face recognition is a considerable challenge for children with and without autism.

## Introduction

Face identity recognition is a complex skill performed rapidly and seemingly effortlessly by mature adults. The ability to discriminate between faces is present very early in development (Field, Cohen, Garcia, & Greenberg, 1984) and improves markedly between early childhood and adolescence (Bruce et al., 2000, Carey et al., 1980, Ellis, 1992, Mondloch et al., 2003). Yet the emergence of adult face expertise follows a protracted developmental trajectory, with performance on tests of unfamiliar face recognition not approaching maturity until well into adulthood (Germine et al., 2011, Susilo et al., 2013). Much research has focused on the mechanisms underlying this lengthy course of development, including holistic, configural, and norm-based coding abilities (Crookes and McKone, 2009, Jeffery et al., 2013, Mondloch et al., 2002, Mondloch et al., 2003, Pellicano and Rhodes, 2003, Pellicano et al., 2006, Taylor et al., 2004, Turati et al., 2004), which continue to be the subject of much debate (McKone, Crookes, Jeffery, & Dilks, 2012).

This research, however, has focused on individuals’ abilities to tell *different* faces apart (referred to here as *between-person* face recognition) or to match an image that differs in one experimentally manipulated way, such as facial expression or viewpoint, to a target image of the same identity (Bruce et al., 2000, Ellis, 1992, Mondloch et al., 2003, Sporer et al., 2007). Consequently, existing research cannot explain how we recognize the same “real” face in different contexts (Burton, 2013). Only one identified study has investigated individuals’ ability to recognize the *same* identity across several naturally varying images of the same face (referred to here as *within-person* face recognition). Working on the assumption that muscular movement, lighting, resolution, and depth of contrast can lead to considerable variability in photographs of the same face, Jenkins, White, Van Montfort, and Burton (2011) gave adult participants 20 naturally varying images each of two unfamiliar Dutch celebrities sourced from the Internet and asked them to sort the photographs by identity. No participant arrived at the correct solution of two identities. In fact, the median solution was 7.5 identities, and solutions ranged considerably from 3 to 16. These findings suggest that adults find it extremely challenging to process the wide variability in “real” photographs of the same (unfamiliar) face.

The mechanisms necessary for the difficult task of within-person face recognition are as yet unknown, but it is possible that an averaged representation of facial identity, central to several models of between-face identity recognition (Bruce and Young, 1986, Burton et al., 2005, Valentine, 1991), plays a crucial role. According to norm-based coding models (Leopold et al., 2001, Rhodes and Jeffery, 2006), a viewer creates an internal representation of an average face based on all of the different faces to which that particular individual has been exposed. Accurate between-person face recognition occurs through the positioning of facial identities as vectors from this norm in a multidimensional face space (Valentine, 1991). Burton et al. (2005) further proposed that a “population mean” is calculated for each *separate* facial identity and updated after every encounter to improve reliability. Successful within-person face recognition, therefore, may require a particularly fine-grained norm-based coding system in which an averaged internal representation is created separately for each facial identity. New images of the same face may then be coded as deviations from that particular identity’s “norm.” If judged to be close enough in multidimensional face space, commonalities extracted from this image may then be incorporated into this normed representation.

Bruce (1994) suggested that it is continuous exposure to faces from various viewpoints, lighting conditions, and angles that helps us to derive stable averaged representations of faces through variation. According to Burton et al. (2005), these internalized representations adopt only the elements of identity that are consistent across many viewings (e.g., the structural aspects of the face such as the spatial relationship between the eyes and nose or between the nose and mouth) and discard the more superficial and changeable aspects of a face (e.g., haircut, lighting, expression). Jenkins et al.’s (2011) task, therefore, may be a particularly challenging one because it may require an ability to create and continually update an internalized representation of a new facial identity, reliably distilling the stable elements of identity within a series of 40 encountered images of only two unfamiliar people.

Studies investigating individuals’ ability to distinguish different identities suggest that a norm-based face space is present in young children, including those as young as 4 years (Jeffery et al., 2013, Nishimura et al., 2008, Pimperton et al., 2009). In fact, different responses to average faces, as opposed to “distinct faces,” in infants (Rhodes, Geddes, Jeffery, Dziurawiec, & Clark, 2002) suggest that it could be present even earlier. Whereas Crookes and McKone (2009) argued that general cognitive improvements in the visual system or in working memory are likely candidates for the mechanisms underlying age-related improvements in face identity recognition, Jeffery et al. (2013) suggested that the quantity and quality of dimensions of face space may undergo fine-tuning during development, leading to a more efficient and precise face identification system.

To date, all of this research has examined norm-based coding within the context of between-person face recognition. There is no study examining within-person recognition of unfamiliar faces in different contexts in children. As challenging as it is for adults, Jenkins et al.’s (2011) task may be especially difficult for children because it may require participants to create and update an average of two facial identities repeatedly in a short space of time, extract the commonalities between images, and build a stable representation “online.”

Within-person face recognition might also be particularly challenging for children diagnosed with autism, a neurodevelopmental condition that affects the way an individual interacts with and experiences the world around him or her (American Psychiatric Association [APA], 2013). Atypicalities in face processing are well documented in individuals on the autism spectrum (Rutherford et al., 2007, Wallace et al., 2008, Wilson et al., 2012, Wolf et al., 2008), including greater difficulty in recognizing unfamiliar faces in autistic children compared with children of similar age and ability (Boucher and Lewis, 1992, Klin et al., 1999). There have also been swathes of studies that failed to find any such differences. Indeed, Weigelt, Koldewyn, and Kanwisher (2012) reviewed 90 studies, of which only half found that autistic people perform worse than typical individuals (*n* = 46), with the other half finding no difference (*n* = 44), although they concluded that autistic individuals had difficulties in tasks specifically involving face memory and in discriminating eye gaze.

Researchers investigating the source of potential difficulties in face recognition in autism have implicated weaker norm-based coding of facial identity (Ewing et al., 2013, Fiorentini et al., 2012, Pellicano et al., 2007, Rhodes et al., 2014). These experimental findings are consistent with a recent theoretical account that situates autistic perception within a Bayesian framework. According to Pellicano and Burr (2012), autistic individuals are less likely to use prior information to interpret incoming sensory information and, therefore, have difficulties in discerning the salience of incoming sensory information. In face identity recognition, less reliance on prior knowledge (i.e., knowledge accrued with experience) may translate to difficulties in discarding information that is irrelevant to identity judgments (e.g., the superficial and changeable aspects of facial images) and identifying the commonalities over repeated viewings necessary for face identification according to Bruce, 1994, Burton et al., 2005 models. If autistic individuals have difficulty in creating or updating an abstract representation of a person’s face, they should have difficulty in recognizing several unfamiliar images of the same person as belonging to the same face.

### The current study

The aims of this study were twofold. First, we sought to extend research on within-person face recognition using multiple images of the same identity to children and investigate age-related changes in this ability. In Experiment 1, we modified Jenkins et al.’s (2011) procedure to ensure that it was engaging and developmentally appropriate for children between 6 and 14 years of age. Children were shown 40 naturally varying photographs of two different male faces and were asked to sort them by identity. We predicted that, in line with other findings on the development of *between-person* face identity recognition, younger participants would perform less well than older participants, perceiving a greater number of identities among the 40 images of the two men than older participants.

Second, we investigated the within-person face recognition skills of children with and without autism. In Experiment 2, we compared autistic children’s task performance with a subgroup of children from Experiment 1 matched for age and intellectual ability. We predicted that children on the autism spectrum would have difficulties in developing stable internal representations of newly presented unfamiliar identities (cf. Pellicano & Burr, 2012) incommensurate with their age and ability.

## Experiment 1

### Method

#### Participants

A total of 77 children participated in this experiment, including 16 6- and 7-year-olds (*M* = 6;10 [years/months], range = 6;0–7;11, 6 girls), 28 8- and 9-year-olds (*M* = 9;3, range = 8;2–9;11, 9 girls), 20 10- and 11-year-olds (*M* = 10;11, range = 10;1–11;11, 14 girls), and 13 12- to 14-year-olds (*M* = 13;2, range = 12;0–14;9, 11 girls). One additional child was tested but excluded from analysis for not understanding task instructions (see below). In addition, 15 adults between 26 and 37 years of age also participated (*M* = 31;1, 6 women). Participants were recruited through mainstream schools and community contacts in the Greater London area.

#### Stimuli

**Fig. 1:**
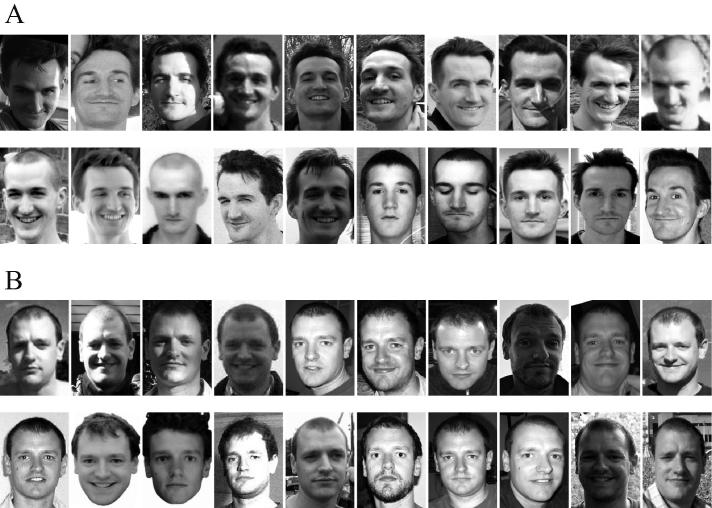
Face stimuli used in Experiments 1 and 2. A shows the twenty images of identity 1 (Rob). B shows the twenty images of identity 2 (Dom).

Participants were presented with 40 laminated grayscale photographs of two male identities (20 of “Rob” and 20 of “Dom”) measuring 85 × 65 mm each. The individuals in the photographs were unfamiliar to participants. Because this study sought to examine children’s responses to naturally occurring variability in images of faces, we did not use experimentally manipulated images. Rather, and following Jenkins et al. (2011), the images encompassed a diverse range of natural photographs taken of the identities at different ages, from different angles, and in different lighting conditions. Photographs showed faces in front view with no obstructions. The images used can be seen in Fig. 1.

#### Procedure

Participants were seen individually in a quiet room at their school, their place of work, or the university. For all participants—children and adults—the task was presented within the context of a game, with a cover story about a detective who needed their help to identify criminal suspects. Next, participants were presented with the 40 grayscale images in a shuffled deck and were instructed to sort the photographs into piles by identity. Specifically, participants were instructed to place photographs of the same person together and to place photographs of different people into different piles, so that they ended up with a separate pile for each different person. Participants were asked to repeat the task instructions to the experimenter in their own words before proceeding so that the experimenter could be confident that participants fully understood them. Participants were given 10 min to complete the task. Pilot testing suggested that this time limit was adequate. Indeed, during the experiment proper, the majority of participants (including half of 6- and 7-year-olds) did not make use of all the time available to them, indicating to the experimenter that they had finished the task well before reaching the time limit. Following Jenkins et al. (2011), no further instructions were given and no feedback was provided regarding accuracy. Photographs were numbered on the reverse from 1 to 40, and the experimenter used these numbers to record which photographs had been placed in each pile. The number of identities perceived by each participant (maximum possible = 40) and the number of misidentification errors (where the two different identities were perceived to be the same identity) were the dependent variables of interest.

This study was granted ethical approval by the Institute of Education’s research ethics committee. Written informed consent was obtained by participating adults and from parents prior to their children’s participation in this study. Children gave their verbal assent to take part.

### Results

#### Number of perceived identities

**Fig. 2:**
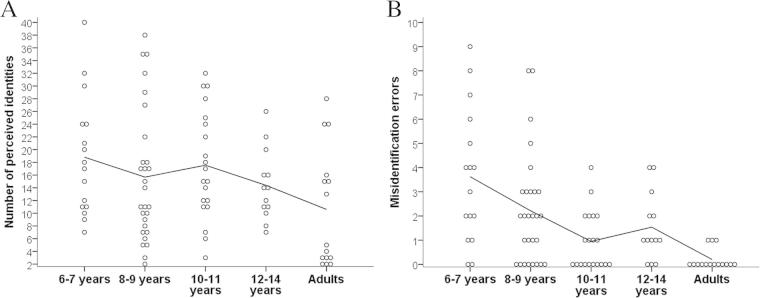
Scatterplots showing the number of perceived identities (A) and the number of misidentification errors (the number of times a participant’s image piles featured more than one real identity) (B) for each participant by age group. Lines indicate changes in mean scores across development.

Descriptive statistics are reported in Table 1. Consistent with Jenkins et al. (2011), adults (*n* = 15) often mistook images of the same person as images of different people, subdividing Rob and Dom into many perceived identities (median = 5, range = 2–28). Children (*n* = 77) fractionated the identities even further (median = 14.5, range = 2–40), frequently judging images of the same identity to be too dissimilar to go together. Two adults and none of the children arrived at the correct solution. Performance on the task varied considerably within each age group, as can be seen in Fig. 2A.

To investigate age-related differences in the number of perceived identities further, we carried out a mixed-design analysis of variance (ANOVA) with age group (6- and 7-year-olds, 8- and 9-year-olds, 10- and 11-year-olds, 12- to 14-year-olds, or adults) as the between-participants factor and identity (Rob or Dom) as the within-participants factor. There was a significant effect of age, *F*(1, 87) = 3.80, *p* = .01, *η*_p_^2^ = .15. Planned comparisons revealed that adults (*M* = 10.60, *SD* = 9.28) divided images into significantly fewer groups than both 6- and 7-year-olds (*M* = 18.81, *SD* = 9.34), *t*(29) = 2.45, *p* = .02, *d* = 0.88, and 10- and 11-year-olds (*M* = 17.55, *SD* = 8.51), *t*(33) = 2.30, *p* = .03, *d* = 0.78. There were no other significant differences (all *p*s > .10). There was a significant main effect of identity, *F*(1, 87) = 5.33, *p* = .02, *η*_p_^2^ = .06, with images of Rob divided into significantly fewer piles (*M* = 8.30, *SD* = 4.72) than Dom (*M* = 9.07, *SD* = 4.82). There was no significant identity × age group interaction, *F*(4, 87) = 5.33, *p* = .88, *η*_p_^2^ = .01.

#### Misidentification errors

In Jenkins et al.’s (2011) study, poor performance was principally a failure to unify images of the same identity, with adults unlikely to make any misidentification errors (placing images of two different identities in the same pile). To assess whether the same was true in our developmental sample, each of an individual’s “perceived identities” (image piles) was assigned a “0” if it (correctly) featured images of only one identity (i.e., either Rob or Dom) and a “1” if it featured images of both identities (i.e., Rob *and* Dom). These scores were summed to create a total error score for each participant (maximum score = 20). Similar to Jenkins and colleagues, misidentification errors among adults were rare (median = 0, range = 0–1), but these errors were more common in children (median = 2, range = 0–9) (see Table 1). The numbers of misidentification errors varied considerably among the childhood age groups (see Fig. 2B).

To investigate developmental effects, a one-way ANOVA was performed on misidentification errors. There was a significant effect of age group, *F*(4, 87) = 7.86, *p* < .001, *η*_p_^2^ = .27. Planned comparisons suggested that, broadly, misidentification errors decreased with age. Although 6- and 7-year-olds (*M* = 3.63, *SD* = 2.78) did not make significantly more errors than 8- and 9-year-olds (*M* = 2.21, *SD* = 2.28), *p* = .08, *d* = 0.56, they did make significantly more errors than 10- and 11-year-olds (*M* = 0.95, *SD* = 1.20), *t*(34) = 3.89, *p* < .001, *d* = 1.25, 12-to 14-year-olds (*M* = 1.54, *SD* = 1.39), *t*(27) = 2.46, *p* = .02, *d* = 0.95, and adults (*M* = 0.20, *SD* = 0.41), *t*(29) = 4.72, *p* < .001, *d* = 1.73. In addition, 8- and 9-year-olds made significantly more errors than 10- and 11-year-olds, *t*(42.69) = 2.49, *p* = .02, *d* = 0.69, and adults, *t*(41) = 3.37, *p* = .002, *d* = 1.23. Furthermore, 10- and 11-year-olds and 12- to 14-year-olds made more errors than adults (*p* = .02, *d* = 0.84 and *p* = .005, *d* = 1.31, respectively).

#### Matrix analysis

**Fig. 3:**
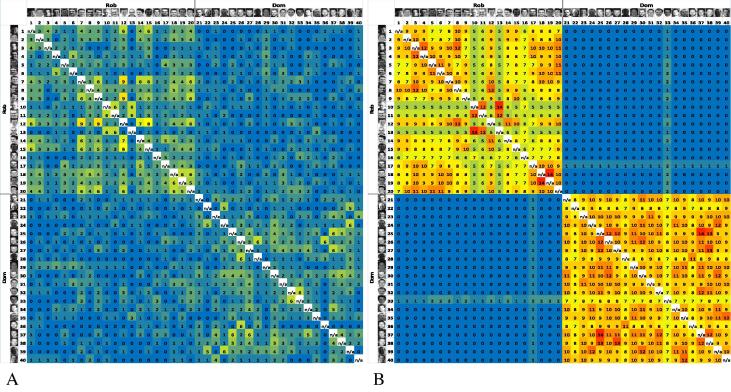
In these 40 × 40 matrices, all 40 images (1–20: Rob; 21–40: Dom) are placed along both the *x* and *y* axes. The value in each cell specifies the number of times two images were placed in the same pile pooled across 16 6- and 7-year-olds (A) and 15 adults (B). Therefore, values reflect the number of participants placing the two images together in the same pile. Perfect performance would result in 16 s (for children) or 15 s (for adults) in the top-left and bottom-right quadrants and 0 s in the top-right and bottom-left quadrants. Non-zero values in the latter two quadrants represent identity merge errors, that is, photos of different people being grouped together. Cell values are highlighted in a blue (low scores) to yellow and then red (high scores) color gradient. Because an image cannot be matched with itself, blank cells run in a diagonal line from the top-left to bottom-right corners of the matrices.

Neither of the two outcome measures described so far fully characterized task performance. A participant could receive a perfect score for the number of perceived identities (two) even if both of the participant’s identity piles featured Rob and Dom. Similarly, a participant could receive a perfect score for misidentification errors (zero) even if the participant sorted the 40 images into 40 different identity piles. A series of matrices, therefore, was created to visualize task outcomes and to derive an integrated measure of task performance (see Fig. 3). All 40 images (1–20 of Rob and 21–40 of Dom) were placed along both the *x* and *y* axes, yielding 400 cells to record incidences where two images of different identities were placed together (20 Rob × 20 Dom) and 760 cells to record incidences where two images of the same identity were placed together (380 Rob × Rob and 380 Dom × Dom) for each participant separately. The 92 performance matrices (one for each participant) were then combined into five “age-binned” performance matrices to compare patterns of performance across groups. The performance matrices for 6- and 7-year-olds (*n* = 16) and adults (*n* = 15) are illustrated in Fig. 3A and B, respectively. All five age-binned matrices can be seen in the online supplementary material.

Each performance matrix had four “quadrants.” The top-left and bottom-right quadrants show the number of times participants correctly matched images of Rob/Dom with other images of Rob/Dom. The top-right and bottom-left quadrants, which are symmetrical across the diagonal, show the number of times participants *incorrectly* placed images of Dom with images of Rob. For 6- and 7-year-olds (*n* = 16), therefore, perfect performance would result in a value of “16” in every cell in the top-left and bottom-right quadrants (same identity match) and a value of “0” in every cell in the top-right quadrant and its duplicate in the bottom-left quadrant.

To compare groups on an integrated measure of task performance, we first calculated a “match score” by summing the values in the top-left and bottom-right quadrants for each participant separately and a “mismatch score” by summing the values in the top-right quadrant for each participant separately. Each participant’s match score was then divided by his or her mismatch score to yield a “matrix score,” a ratio of correct sorting to incorrect sorting for each participant. A constant of 1 was added to each participant’s mismatch score beforehand to ensure that all mismatch scores were non-zero. Because of the large range in scores, and violations to the assumption of homogeneity of variance, nonparametric analyses were used on the matrix score. A Kruskal–Wallis nonparametric test on matrix scores revealed a significant effect of age group, *H*(4) = 39.97, *p* < .001 (see Table 1 for scores; higher scores indicate better performance). Mann–Whitney *U* tests further revealed that 6- and 7-year-olds performed significantly worse than 8- and 9-year-olds (*U* = 308.0, *z* = 2.05, *p* = .04, *r* = .31), 10- and 11-year-olds (*U* = 267.0, *z* = 3.41, *p* < .001, *r* = .57), 12- to 14-year-olds (*U* = 167.0, *z* = 2.76, *p* = .005, *r* = .51), and adults (*U* = 236.0, *z* = 4.59, *p* < .001, *r* = .82). In addition, 8- and 9-year-olds performed significantly worse than 10- and 11-year-olds (*U* = 382.0, *z* = 2.13, *p* = .03, *r* = .31) and adults (*U* = 393.0, *z* = 4.66, *p* < .001, *r* = .71), whereas adults also performed better than 10- and 11-year-olds (*U* = 268.5, *z* = 3.95, *p* < .001, *r* = .67), and 12- to 14-year-olds (*U* = 184.0, *z* = 3.99, *p* < .001, *r* = .76).

Overall, the results suggest that within-person face recognition is a particularly challenging task, especially for children. Analyses on the number of perceived identities showed significant improvements in performance between young children and adults. Misidentification errors also decreased with age. A more fine-grained analysis using performance matrices showed a general pattern of age-related improvements, with adults performing better than older children, who in turn performed better than younger children on a summary performance measure.

These findings suggest that the ability to integrate successfully different images of the same face into a single representation of facial identity follows a lengthy developmental trajectory, just like between-person face recognition (Germine et al., 2011, Susilo et al., 2013).

## Experiment 2

### Method

#### Participants

A total of 32 6- to 14-year-old children diagnosed with autism (*M*_age_ = 11;1, *SD* = 2;7, 5 girls) were recruited through advertisements, the Autism Spectrum Database–UK (http://www.ASD-UK.com), mainstream and special schools, and parent support groups in the Greater London area. All children had an independent clinical diagnosis of an autism spectrum condition according to DSM-IV (*Diagnostic and Statistical Manual of Mental Disorders,* 4th edition) criteria (APA, 1994). Parents completed the Social Communication Questionnaire (SCQ; Rutter, Bailey, & Lord, 2003), and autistic children were administered the Autism Diagnostic Observation Schedule (ADOS-G or ADOS-2; Lord et al., 1999, Lord et al., 2012) using the revised algorithm (Gotham et al., 2007, Gotham et al., 2008). All children with autism scored above the threshold for an autism spectrum condition on one or both of these diagnostic measures. An additional 5 autistic children were assessed but excluded from the dataset either because there was uncertainty over whether they understood the instructions (*n* = 4) or because they had a full-scale IQ score below 70, as measured by the Wechsler Abbreviated Scales of Intelligence–Second Edition (WASI-II; Wechsler, 2011) (*n* = 1).

A subgroup of 32 typically developing children (*M*_age_ = 10;7, *SD* = 2;2, 12 girls) who participated in Experiment 1 was matched individually with the autistic children for age and cognitive ability. There were no significant group differences in terms of chronological age, *t*(62) = 0.84, *p* = .40, verbal IQ, *t*(62) = 1.12, *p* = .27, performance IQ, *t*(59.25) = 0.34, *p* = .74, or full-scale IQ, *t*(62) = 0.87, *p* = .38, as measured by the WASI-II (see Table 2 for scores). Given that there were no differences between males and females’ performance in Experiment 1 in terms of the number of perceived identities (*p* = .24), misidentification errors (*p* = .18), or the matrix total scores (*p* = .32), the autism and typical groups were not matched on gender. Of the children whose parents completed the SCQ, no child scored above the cutoff of 15 for autism specified by Rutter and colleagues (2003), indicative of low levels of autistic symptomatology.

#### Procedure

The stimuli and procedure were identical to those in Experiment 1. Informed consent was obtained from the parents of all children prior to participation, and verbal assent was obtained from participating children.

### Results

#### Number of perceived identities

**Fig. 4:**
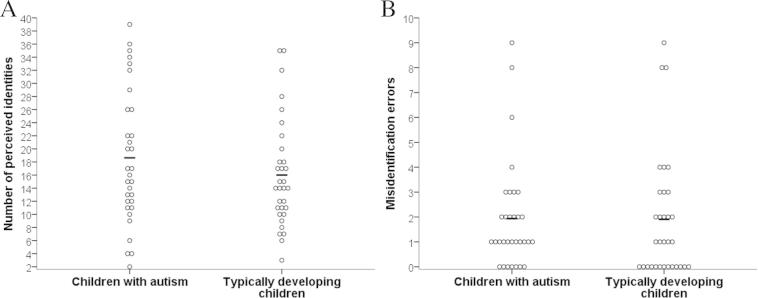
Scatterplots showing the number of perceived identities (A) and the number of misidentification errors (the number of times a participant’s image piles featured more than one real identity) (B) for autistic and typically developing children. Markers indicate mean scores.

Descriptive statistics for autistic and typical children are reported in Table 3. Autistic children sorted images into a median of 16.5 piles, and typical children sorted images into a median of 14 piles. As can be seen in Fig. 4A, there was wide variability in performance within groups, with the number of perceived identities ranging from 2 to 39 in the autism group and from 3 to 35 in the typical group. A mixed-design ANOVA on the number of perceived identities with identity (Rob or Dom) as the within-participants factor and group (autism or typical) as the between-participants factor revealed, unexpectedly, no main effect of group, *F*(1, 62) = 1.45, *p* = .23, *η*_p_^2^ = .02. There was also no main effect of identity, *F*(1, 62) = 2.84, *p* = .10, *η*_p_^2^ = .04, and no significant identity × group interaction, *F*(1, 62) = 0.90, *p* = .35, *η*_p_^2^ = .02.

#### Misidentification errors

An independent-sample *t*-test on children’s misidentification errors (the number of their image piles featuring more than one identity) revealed similar numbers of errors in autistic (*M* = 1.94, *SD* = 2.18) and typical children (*M* = 1.91, *SD* = 2.49), *t*(62) = 0.05, *p* = .96, *d* = 0.01. Again, there was a large amount of variance within each group, with scores ranging from 0 to 9 in children with and without autism (see Fig. 4B).

#### Matrix analysis

**Fig. 5:**
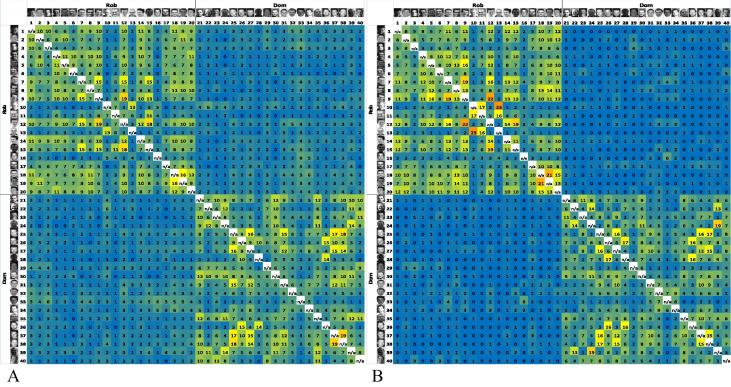
In these 40 × 40 confusion matrices, all 40 images (1–20: Rob; 21–40: Dom) are placed along both the *x* and *y* axes. The value in each cell specifies the number of times two images were placed in the same pile pooled across the 32 autistic children (A) and the 32 typically developing children (B). Perfect performance would result in 32 s in the top-left and bottom-right quadrants and 0 s in the top-right and bottom-left quadrants. Non-zero values in the latter two quadrants represent identity merge errors, that is, photos of different people being grouped together. Cell values are highlighted in a blue (low scores) to yellow and then red (high scores) color gradient. Because an image cannot be matched with itself, blank cells run in a diagonal line from the top-left to bottom-right corners of the matrices.

Similar to Experiment 1, performance matrices plotting the number of times each of the 40 images was placed in a pile with each of the other 39 images were created for each autistic child (*n* = 32) and each typical child (*n* = 32). The values in these matrices were pooled to create two “group-binned” matrices, shown in Fig. 5. For autistic children (Fig. 5A), the values in the top-left and bottom-right quadrants ranged from 0 to 19, meaning that the number of times two images of Rob/Dom were correctly placed together ranged from never (0%) to 19 (59%). For typically developing children (Fig. 5B), the values in these same quadrants were largely similar, ranging from never (0%) to 23 (72%).

Next, values in the top-right quadrant (duplicated in the bottom-left quadrant), which represent the number of times two images of different identities were incorrectly perceived as being the same person, were compared across groups. The values in these quadrants ranged from 0 to 6 in both groups, meaning that at most 6 children (19%) in each group mistakenly placed a pair of mismatched images together.

Finally, and similar to Experiment 1, a total matrix score was calculated for each participant by dividing each child’s match score (the sum of the values in the top-left and bottom-right quadrants) by the child’s mismatch score (the sum of the values in the top-right quadrant) As before, a constant of 1 was added to each participant’s mismatch score beforehand to ensure that all mismatch scores were non-zero. Descriptive statistics on these matrix scores are shown in Table 3. A Mann–Whitney *U* test on children’s matrix scores revealed a marginally significant group difference (*U* = 370.5, *z* = –1.90, *p* = .057, *r* = –.24). These results suggest that children with autism have similar difficulties as typically developing children in recognizing the same facial identity across several images, with a trend toward poorer performance on the task overall.

## General discussion

Developmental improvements in face identity recognition are well documented. Although research has focused on same–different judgments of experimentally manipulated images, so far it has not considered individuals’ abilities to recognize the same identity across *multiple* viewings within a developmental context. In this study, an experimental task designed to test how well individuals recognize differing and naturally varying images of the same identity was extended to children for the first time. Similar to Jenkins et al. (2011), we found that within-person face recognition is a difficult task for adults, with only 2 of 15 participants arriving at the correct solution. Our findings further showed, however, that it is an exceptionally difficult task for children. None of the 77 children between 6 and 14 years of age arrived at the correct solution. Furthermore, we observed that misidentification errors, a rarity among adults, were much more common in young children. This means that as well as failing to recognize the same face across different contexts, children were also more likely to perceive different identities as being the same identity.

These findings add to the wealth of existing research suggesting that face identity recognition follows a protracted developmental course (Germine et al., 2011, Mondloch et al., 2003, Susilo et al., 2013). Age differences in the overall number of identities perceived, and the number of times images of the same identity were placed together, indicate that within-person face identity recognition does not improve significantly within middle childhood but does improve significantly between childhood and adulthood. The number of misidentification errors appeared to follow a slightly clearer developmental path. Younger children made significantly more errors than older children, and older children made more errors than adults, who made almost no misidentification errors at all.

One way of interpreting these findings is to view a greater number of identities perceived as a failure of within-person face recognition (a failure to recognize that several images are of the same identity) and higher numbers of misidentification errors as a failure of between-person face recognition (a failure to recognize two different identities as being separate). Working on the assumption that generally there is a greater difference between two images of different identities than between two images of the same identity, an ability to discriminate between two unfamiliar identity images might emerge earlier in development, with errors decreasing as the basic structure of “multidimensional face space” (Valentine, 1991), present from a young age (Jeffery et al., 2013, Nishimura et al., 2009, Nishimura et al., 2008, Pimperton et al., 2009), undergoes adjustments and improvements (Jeffery et al., 2013). An arguably more sophisticated system, which can efficiently carry out between-person face recognition, may be a necessary requirement for within-person recognition as measured by the current task. This sophistication may involve the abilities to weight or integrate information from multiple dimensions, which children appear to be less skilled at or inclined to do (Nishimura et al., 2009). Within a norm-based coding model, success on the current task depends on a viewer positioning images much closer together in face space, as vectors from the “norm” of that particular individual’s identity, before deciding whether to integrate that image into the averaged representation for that face.

Using the concept of “stability through variation” (Bruce, 1994), Burton et al. (2005) proposed that an internal representation, or “population mean,” of individual facial identities (e.g., Dom) is readjusted after each new encounter to improve reliability. If we follow this model, in Jenkins et al.’s (2011) task, participants must begin to build this internal representation from scratch and then update it repeatedly as they sort through the 40 images and decide which ones to encode as a representation of an existing identity and incorporate into that population mean and which ones to keep separate. In accordance with this norm-based coding model, this particular identity’s vector in multidimensional face space must be continually adjusted in comparison with that person’s concept of an average face. The larger the variance among images of the same face, the more sophisticated this system needs to be to extract the commonalities between them. This may explain why an ability to recognize the same identity across several different images remains so difficult even for adults.

We also examined for the first time within-person face recognition in autistic children. Our findings showed no clear differences between children with and without autism, of similar age and ability, on the number of identities perceived or on the number of misidentification errors made (image piles featuring both identities). Although this is not the only study to report similar performance between autistic and typically developing individuals on a face processing task (Yi et al., 2015), difficulties in face identity recognition are well documented in autism (Dawson et al., 2005, Weigelt et al., 2012), and so these findings might appear to be somewhat surprising. One possible explanation for these null findings is the considerable variability in children’s performance within each group, which may have precluded the possibility of detecting group differences in within-person face recognition in Experiment 2 (and indeed of detecting such age-related differences in Experiment 1). Alternatively, the difficulty of the task may have also prevented us from detecting differences in performance by children with and without autism. Future research should examine within-person face recognition in children with and without autism using a simpler task—perhaps by using fewer images to sort—or by assessing such skills in adolescents or adults (rather than children) with and without autism, when such abilities are more mature and potentially vary less between individuals.

One further possibility relates to the nature of the task itself. Successful overall performance on the task requires an ability to tell different faces apart *as well as* an ability to tell faces together. Our integrated measure of task performance reflecting these abilities did reveal a marginally significant difference between the groups. One possible interpretation of a trend toward reduced overall performance in autism is difficulties in building stable “averaged” representations of a “normed” facial identity (Leopold et al., 2001, Rhodes and Jeffery, 2006, Valentine, 1991). Evidence from the face identity aftereffect task suggests that there may be reduced updating of face norms in response to experience in children with autism (Ewing et al., 2013, Pellicano et al., 2007) and in relatives of those with autism (Fiorentini et al., 2012). This is in line with a theory of autistic perception (Pellicano & Burr, 2012), which suggests that autistic individuals are less likely to use prior information to interpret incoming sensory information. Such a bias could make it difficult to discard information that is somewhat irrelevant to identity judgments (e.g., superficial and changeable aspects of facial images such as hairstyle, head angle, and lighting), increasing the likelihood of two images of the same identity being judged as different but also of two images of different identities being judged as the same. Caution is warranted, however, when interpreting this marginal result. Further replication of the result reported here is necessary before strong conclusions can be drawn.

One strength of this study is that it used naturally varying images—“real photos”—unlike the vast majority of experimental studies where images of faces are often manipulated extensively and, therefore, vastly different from images of faces seen outside of the laboratory (Burton, 2013). Yet, even the stimuli used here are devoid of context, which should help individuals to build representations and guide their decisions in everyday life. The use of natural images in an experiment also brings considerable challenges because the substantial variance in the images makes it difficult to isolate underlying mechanisms responsible for this seemingly complicated task. In addition, the task itself was an “open” task, which means that participants could employ a range of different strategies to solve it. Creating and updating an abstract representation of each individual’s facial identity, therefore, is just one possible explanation for successful performance on the task; individuals may have also matched images based on featural or configural information. The extension of eye-tracking research to studies using more naturally varying images has the potential to yield insights into which strategies participants use for successful within-person face recognition by identifying which aspects of the face are attended to in order to perform the task. Further research could also use nonsocial stimuli to see whether these findings are selective to faces, for example, by comparing performance on recognizing several images of the same face with how well participants recognize several images as being of the same object (e.g., cars). Such research will be important in determining whether the age-related improvements found here are task specific, perhaps attributable to the large number of images participants were required to sort and keep track of.

In conclusion, this study has demonstrated the considerable challenge posed by within-person face recognition for typical children, adults, and autistic children alike, and it highlights the need for greater understanding of the mechanisms that underlie this largely overlooked aspect of face perception. This work also has practical implications for working with children, especially children with autism. Photographs of adults supporting autistic children are commonly used in social stories and other tools designed to prepare the children to meet new people or to engage in new activities. The findings here suggest that some children may well struggle to recognize the same people they meet in person from an individual photograph.

## Figures and Tables

**Table 1:** Descriptive statistics by age on performance measures.

	6–7 years	8–9 years	10–11 years	12–14 years	Adults
*Number of perceived identities*^a^*(max = 40)*					
Mean (*SD*)	18.81 (9.34)	15.68 (10.42)	17.55 (8.51)	14.38 (5.58)	10.60 (9.28)
Median	17.5	12.5	16	14	5
Range	7–40	2–38	3–32	7–26	2–28
*Misidentification errors*^b^					
Mean (*SD*)	3.63 (2.78)	2.21 (2.28)	0.95 (1.19)	1.54 (1.39)	0.20 (0.41)
Median	3.5	2	0.5	1	0
Range	0–9	0–8	0–4	0–4	0–1
*Matrix score*^c^					
Mean (*SD*)	13.76 (22.66)	61.08 (136.81)	63.92 (75.09)	43.17 (48.74)	433.20 (296.76)
Median	3.08	10.48	29.50	18.25	518.00
Range	0–84	1.97–562	5.17–252	4.33–142	46–760

a Number of perceived identities is the number of image piles (each representing a different perceived identity) that participants sorted the 40 photographs into within the 10-min time limit.

b Misidentification errors is the number of times participants’ image piles featured more than one real identity (i.e., both Dom and Rob).

c Matrix score is the number of times two images of the same real identity were placed together (as recorded in the top-left and bottom-right matrix quadrants) divided by the number of times two images of differing identities were placed together (as recorded in the top-right matrix quadrant). Higher scores indicate better performance.

**Table 2:** Characteristics of autistic and typically developing children in Experiment 2.

Measure	Children with autism (*n* = 32)	Typically developing children (*n* = 32)
*Age (years;months)*		
Mean (*SD*)	11;1 (2;7)	10;7 (2;2)
Range	6;4–14;8	6;7–14;2
*Verbal IQ*^a^		
Mean (*SD*)	97.47 (15.29)	101.34 (12.06)
Range	71–130	79–131
*Performance IQ*^a^		
Mean (*SD*)	100.13 (15.51)	101.31 (12.46)
Range	75–128	74–128
*Full-scale IQ*^a^		
Mean (*SD*)	98.59 (14.85)	101.50 (11.38)
Range	70–128	85–130
*SCQ*^b^		
Mean (*SD*)	26.63 (6.50)^d^	5.43 (3.81)^e^
Range	16–45	0–14
*ADOS*^c^		
Mean (*SD*)	8.93 (2.51)^f^	
Range	6–16	

a Verbal IQ, performance IQ, and full-scale IQ were all measured using the WASI-II (Wechsler, 2011).

b SCQ is the Social Communication Questionnaire. A score of 15 or above indicates elevated levels of autistic symptomology.

c ADOS is the Autism Diagnostic Observation Schedule. Scores of 7 or above indicate the presence of an autistic spectrum condition.

d *n* = 30.

e *n* = 23.

f *n* = 28.

**Table 3:** Descriptive statistics for autistic and typically developing children on performance outcome measures.

Measure	Autistic children (*n* = 32)	Typically developing children (*n* = 32)
*Number of perceived identities*^a^		
Mean (*SD*)	18.63 (10.18)	16.00 (8.21)
Median	16.5	14
Range	2–39	3–35
*Misidentification errors*^b^		
Mean (*SD*)	1.94 (2.18)	1.91 (2.49)
Median	1	1
Range	0–9	0–9
*Matrix score*^c^		
Mean (*SD*)	36.02 (64.28)	69.50 (118.14)
Median	8.88	18.92
Range	1.83–264	1.55–562

a Number of perceived identities is the number of image piles (each representing a different perceived identity) that participants sorted the 40 photographs into within the 10-min time limit.

b Misidentification errors is the number of times participants’ image piles featured more than one real identity (i.e., both Dom and Rob).

c Matrix score is the number of times two images of the same real identity were placed together (as recorded in the top-left and bottom-right matrix quadrants) divided by the number of times two images of differing identities were placed together (as recorded in the top-right matrix quadrant). Higher scores indicate better performance.

## Appendix A Supplementary data

Supplementary Fig. 6 In these five 40 × 40 performance matrices all 40 images (1–20: Rob and 21–40: Dom) are placed along both the *x* and *y* axes. The value in each cell specifies the number of times two images were placed in the same pile pooled across 6- and 7-year-olds (A), 8- and 9-year-olds (B), 10- and 11-year-olds (C), 12- to 14-year-olds (D) and adults (E). The top left and bottom right quadrants show correct same identity matches, whereas the top right and bottom left quadrants (duplicates of one another) show incorrect, different identity matches. Cell values are highlighted in a blue (low scores) to yellow then red (high scores) color gradient. Perfect performance by the group would result in entirely red top left and bottom right quadrants, and entirely blue top right and bottom left quadrants.
