# Supplementary material for: Recognizing the same face in different contexts: Testing within-person face recognition in typical development and in autism
Source: J Exp Child Psychol. 2016 Mar;143:139–53. doi: 10.1016/j.jecp.2015.09.029 (PMC4722798; doi:10.1016/j.jecp.2015.09.029)
Supplement: Supplementary Fig. 6 — In these five 40 × 40 performance matrices all 40 images (1–20: Rob and 21–40: Dom) are placed along both the x and y axes. The value in each cell specifies the number of times two images were placed in the same pile pooled across 6- and 7-year-olds (A), 8- and 9-year-olds (B), 10- and 11-year-olds (C), 12- to 14-year-olds (D) and adults (E). The top left and bottom right quadrants show correct same identity matches, whereas the top right and bottom left quadrants (duplicates of one another) show incorrect, different identity matches. Cell values are highlighted in a blue (low scores) to yellow then red (high scores) color gradient. Perfect performance by the group would result in entirely red top left and bottom right quadrants, and entirely blue top right and bottom left quadrants. [file mmc1.docx]

**Figure 6B: 8-9 year olds**

**Figure 6A: 6-7 year olds**

**Figure 6D: 12-14 year olds**

**Figure 6C: 10-11 year olds**

**Figure 6E: Adults**

Figure 3A: 6-7 year olds

*Figure 6*. In these five 40x40 performance matrices all 40 images (1-20: Rob and 21 – 40: Dom) are placed along both the x and y axes. The value in each cell specifies the number of times two images were placed in the same pile pooled across 6- and 7- year-olds (Figure 6A), 8- and 9- year-olds (Figure 6B), 10- and 11- year-olds (Figure 6C), 12- to 14- year-olds (Figure 6D) and adults (Figure 6E). The top left and bottom right quadrants show correct same identity matches, whereas the top right and bottom left quadrants (duplicates of one another) show incorrect, different identity matches. Cell values are highlighted in a blue (low scores) to yellow then red (high scores) color gradient. Perfect performance by the group would result in entirely red top left and bottom right quadrants, and entirely blue top right and bottom left quadrants.
